# Country profile – Primary healthcare and family medicine in Namibia

**DOI:** 10.4102/phcfm.v12i1.2242

**Published:** 2020-01-27

**Authors:** Felicia Christians

**Affiliations:** 1 Department of Family and Community Medicine, Faculty of Health Sciences, University of Namibia, Windhoek, Namibia

**Keywords:** Namibia, health services, primary health care, family medicine, generalist doctors

## Abstract

Namibia is one of the least densely populated countries in Southern Africa. Namibia’s health services are twofold: private (serving 18% of the population with medical aid) and public (serving the remaining 82%). This, in part, is due to the country’s high income inequality. Access to healthcare is comparably good with 76% of the population living within a 10km radius of a healthcare facility. Yet, Namibia faces many challenges related to the provision of patient-centred primary health care (PHC). The provision of competent generalist doctors and family physicians has the potential to address the current health care challenges and priorities. The inclusion of family physicians in PHC teams will further aid such efforts.

## Demographic and health overview

Namibia is one of the largest and least populated countries in Southern Africa, with a population of 2.45 million in 2018 and a surface area of 824 300 km^2^. Namibia is a relatively arid country with the Namib Desert along its west coast and the Kalahari Desert in the east. Because of this, the country has a low population density of three people square kilometre, with large population clusters along the northern border regions, where rainfall is more plentiful.^1^ Namibia has experienced rapid urbanisation, as evidenced by the increase in the proportion of the population living in urban areas from 28% in 1991 to 50% in 2018.

Children under the age of 15 years account for 37% of the population, whilst those between 15 and 24 years represent 20.3%, those between 25 and 54 years represent 34.7%, those between 55 and 64 years represent 4.5% and those 65 years and over represent 3.9%.^2^ The fertility rate stood at 3.2 births per woman in 2017.^2^ The overall life expectancy at birth was 64 years, with the average life expectancy for males at 61 years and 66 years for females.^2^

Namibia is classified as an upper-middle-income country with a gross national income (GNI) per capita (population) of $10 320. Namibia’s total health expenditure as a percentage of gross domestic product (GDP) was the highest together with South Africa for this group of comparable countries in Africa.^3^

## Description of the health and district health systems

Before gaining independence from South Africa in 1990, Namibia’s healthcare system reflected a traditional medical model, which focussed mainly on hospital-based and curative services. Health services were generally poor, and income inequality in Namibia was extreme, as was inequity in access to health services. In response, the newly formed independent government of Namibia made a commitment to health as a fundamental human right and integrated racially divided communities into one healthcare system. Within a few years, the national leadership at the Ministry of Health and Social Services (MoHSS) began reforms to focus on transitioning to a system based on a central role for primary healthcare (PHC).

The MoHSS is the manager and provider of public health services in Namibia. It operates a four-tiered health system, consisting of PHC sites, district hospitals, intermediate hospitals and a referral hospital. Clinics are staffed by nurses and pharmacy technicians or assistants. When a patient’s needs exceed their scope of practice or available resources, clinics refer patients to health centres, which are staffed by doctors, pharmacists and nurses. When patients require more care than can be provided in a primary care setting, they are referred to district hospitals. If a patient needs to be seen by a specialist, district hospitals refer the patient to intermediate hospitals. Finally, intermediate hospitals refer the most medically complex patients to the Central Hospital in Windhoek when necessary.

The private sector is sizeable, with 844 private health facilities, 72% of the doctors and a little less than 50% of registered nurses.^3^

Only 18% of the population is covered by medical aid funds. As a result, the remaining 82% of the population are covered by the public health system or out-of-pocket (OOP) expenditure in the private sector.^3^

Overall access to healthcare in Namibia is good, with 76% of the population living within a 10 km radius of a health facility. On average, in rural areas, there are about 5780 people per PHC clinic and 58 825 people per district hospital. Hospitals, however, suffer from overcrowding and long wait times, as a large number of people bypass clinics and health centres closer to home and go directly to hospitals that are perceived to offer a higher quality of care.^3^

## Strength and weaknesses of the current district health system

Namibia has developed a disseminated healthcare system to meet the geographic spread of its population, which requires a focus on integration and coordination across its four tiers. The growing healthcare demands are complicated by a shortage of workforce, including specialised services. Some progress has been made through the creation of public–private partnerships.

Namibia’s ranking as an upper-middle-income country has contributed to decreasing donor support to national programmes.^3^ The impact of decreasing donor support is more apparent now in the face of the country’s economic downturn. Implementation of key health initiatives, such as surveillance, supplementary immunisation activities, responses to non-communicable diseases and others, is constrained by limited budgetary allocation. Namibia still faces challenges for providing equitable, person-centred PHC. In HIV prevalence, Namibia still ranks as the sixth highest in the world. Similar to many other countries, Namibia is undergoing an epidemiological transition from communicable diseases to non-communicable diseases (NCDs), and for some time will continue to face this double burden of disease as seen with the recent outbreaks of Hepatitis E and the fact that NCDs accounted for 43% of deaths in 2014.^4^ In addition, inequity remains a pressing challenge for Namibia overall. In 2010, Namibia had one of the highest income inequalities in the world, which is reflected in the lingering disparities in health access and outcomes seen across income groups, races and geographic locations.^5^

The MoHSS is experiencing high staff turnover in certain operational areas because of a lack of clear career progression. This results in the ongoing need for training of newly recruited staff. Staff who have retired are not replaced because of limited budgetary allocation. The MoHSS staff are generally overstretched with heavy workloads and demands, which may affect programme delivery.^5^

## Family medicine in Namibia

Namibia became one of the latest countries in sub-Saharan Africa to open its own School of Medicine in 2010. Family Medicine was introduced into the curriculum in 2016 when a family physician joined the School of Medicine as head of the Department of Family and Community Medicine. So far, the medical school has produced three cohorts of doctors, and there is growing interest in postgraduate training in family medicine. The Department is in the process of introducing a 2-year Postgraduate Diploma in Family Medicine (FM) and PHC.

Currently, 33 family physicians are registered with the Health Professions Council of Namibia. The majority obtained their qualification through family medicine programmes based in South Africa. These doctors, who are mostly non-nationals, work for the MoHSS, in private practice or for non-government organisations. The MoHSS is slowly starting to recognise the role family physicians can play in the delivery of quality PHC and has now included family physicians in the district health road map.

## Lessons to be learnt and conclusion

In spite of its small population and good health infrastructure, Namibia still lags behind in the delivery of person-centred, quality PHC. Well-trained generalist doctors and family physicians can lead the way in turning this situation around. Namibia should join the rest of Africa in creating viable frameworks to support health for all through the inclusion of family physicians in PHC teams.^6^
